# Abiotic Factors from Different Ecuadorian Regions and Their Contribution to Antioxidant, Metabolomic and Organoleptic Quality of *Theobroma cacao* L. Beans, Variety “Arriba Nacional”

**DOI:** 10.3390/plants11070976

**Published:** 2022-04-03

**Authors:** Raluca A. Mihai, Pablo A. Landazuri Abarca, Bryan A. Tinizaray Romero, Larisa I. Florescu, Rodica Catană, Anush Kosakyan

**Affiliations:** 1 CICTE, Department of Life Science and Agriculture, Universidad de las Fuerzas Armadas—ESPE, Av. General Rumiñahui s/n y Ambato, Sangolquí 171103, Ecuador; 2 IASA 1, Department of Life Science and Agriculture, Universidad de las Fuerzas Armadas—ESPE, Av. General Rumiñahui s/n y Ambato, Sangolquí 171103, Ecuador; palandazuri@espe.edu.ec; 3 National Institute for Public Health Research of Ecuador—INSPI, Quito 170136, Ecuador; btinizaray@inspi.gob.ec; 4 Institute of Biology Bucharest, Romanian Academy, 060031 Bucharest, Romania; larisa.florescu@ibiol.ro; 5 Institute of Parasitology, Biology Centre, Czech Academy of Sciences, 37005 Ceske Budejovice, Czech Republic; kosakyan@paru.cas.cz

**Keywords:** fine flavor, cocoa, Arriba Nacional, soil nutrients, abiotic factors, antioxidant activity

## Abstract

Fine flavor cocoa is a unique category of cocoa that produces almonds with high aromatic potential and several sensory benefits that make it different from the basic or ordinary cocoas. Ecuador is the world’s leader in the production and export of fine flavor cocoa, responsible for 63% of the world’s total production due to the commercialization of the Arriba Nacional variety, known to possess an intense aroma that is unique in the cocoa world market. Besides its organoleptic specificity, this variety represents a source of important bioactive compounds associated with both sensory and health properties. This study evaluates the influence of an abiotic factor, nutritional soil status, on the phytochemical composition (methylxantines and phenolic compounds), and antioxidant and sensory properties of Arriba variety cocoa beans originating from three different geographical regions of Ecuador. We used the Diagnosis and Recommendation Integrated System (DRIS), Folin–Ciocalteau, high-performance liquid chromatography (HPLC), ABTS free-radical-scavenging activity, the α, α-diphenyl-β-picrylhydrazyl free-radical-scavenging method (DPPH), and Ferric reducing antioxidant power (FRAP) analysis to reveal a significant correlation between Mn ions and total phenolic content, a positive implication of N in methylxanthine composition and antioxidant properties, and the importance of Ca, Mg, and K ions in increasing the flavonoid and anthocyanin content of raw cocoa beans. We showed that these nutritional elements can interfere with the nutraceutical and sensory properties of cocoa beans, as Cu, Mg, and K are correlated with anthocyaninic content, while Fe, Ca, P and Zn influenced the flavonoid content. We underline that the Arriba variety is suitable not only for the production of high-quality chocolate, but also for the increasing worldwide nutraceutical market, generating qualitative and competitive products.

## 1. Introduction

Cultivated cocoa (*Theobroma cacao* L.) from the Malvaceae family is a remarkable cash crop, grown mostly in developing nations from hot regions around the Equator, where climatic conditions are suitable for its growth [1]. Recent archaeological studies of ceramic artifacts suggest that cocoa beans were harvested and consumed more than 5500 years ago, Ecuador being the original home of cocoa [2].

Four principal varieties of cocoa tree are known: Criollo, which possesses disease susceptibility; Nacional, with a fine flavor, from Ecuador; Forastero in the Amazonian region; and a hybrid between Forastero and Criollo, named Trinitario [3]. The most common varieties in the market are represented by Forastero, mainly produced in Western Africa [4]. “Fine” cocoa flavor is made by some smaller producers. The International Cacao Organization (ICCO) describes “fine cacao” as a cocoa of superior quality with special and distinctive flavors, as well as organoleptic, chemical, and physical properties. This kind of cocoa represents the top-quality cocoas, and less than 5% of the world’s production [5].

The native Ecuadorian cocoa beans, the “Arriba Nacional” (Nacional × Trinitario complex, Arriba flavor) variety, position this country as the most competitive in Latin America within this field, responsible for more than 62% of the global production of fine cocoa flavor, standing out as the first exporter of this emblematic product. In addition, the climate and the geology of different regions in the country lend themselves to the growth of some of the best cocoa beans [6].

The Ecuador’s fine cocoa is characterized as having “a floral profile with blackcurrants and spice” [7]. The differences in the unique potential flavor character of the Arriba bean may be assigned to the composition and concentration of phytochemicals, which are influenced by botanical origin, location of growth, time of sunshine and rainfall, soil nutrient, ripening, and harvesting [8].

This distinctive flavor is due to the phytochemical bean composition represented by polyphenol compounds such as flavonoids, anthocyanins, flavones, phenols (clovamide and deoxyclovamide), hydroxylated stilbene derivatives (trans-resveratrol and trans-piceid) and phenolic acids [9,10]. Along with these polyphenols, methylxanthines (principally theobromine and caffeine) are other compounds that contribute to the bitter and astringent taste.

Because of its characteristic chemical composition, which determines the famously fragrant aroma and complexity, the raw Nacional Arriba cocoa beans are used for manufacturing cocoa powder, chocolate, and other derived products, which are widely consumed around the world [6,9]. The bioactive compounds (polyphenols and alkaloids) occur commonly in raw beans, or are synthesized during the technological cocoa process, and are not only important for their fragrance and flavor but also for exhibiting different positive effects, such as antioxidant activity, the induction of receptor activity, enzyme inhibition/induction, and gene expression inhibition/induction [7]. Polyphenols are the dominant antioxidant compounds, with a concentration of around 12–18% in the dry weight of raw cocoa beans [9]. Alkaloid caffeine and its catabolic product theobromine contribute to cocoa properties, such as its antioxidant and chemopreventive effects [10]. Some epidemiological studies have associated the flavanol-rich cocoa intake with an ample range of biological effects (anti-inflammatory effects, antiplatelet aggregation, anti-atherosclerotic effects, improved insulin sensitivity, and blood pressure and immune function modulation) [2].

The producers of cocoa and its derivates modify the industrial process and the composition of raw materials, in order to improve their sensory values and positive impact on health. The accumulation of the chemical composition of raw cocoa bean (secondary metabolites) may be dependent on environmental factors (light, temperature, soil water, soil nutrition, and salinity). Generally, variation in environmental factors may alter the content of secondary metabolites [11].

In this paper, we analyzed the secondary metabolites and the antioxidant properties of Arriba cocoa from three distinct areas in Ecuador: the Pacific Coast region, the Amazonian region (which is the main production area in Ecuador), and the Andean region (in the Cotopaxi zone, respectively La Maná-Guasaganda a new promising area). In particular, we focused on the implication of soil nutritive composition on cocoa plant nutritional status and the accumulation of the secondary metabolites on Arriba cocoa beans, such as polyphenols (flavonoids, anthocyanins, proanthocyanidins, and stilbenes) and alkaloids (caffeine and theobromine), bioactive compounds responsible for the unicity of this variety in flavor and a possible source of nutraceutical products.

Taking into account that the total demand of cocoa beans is increasing in the world [12], new studies concerning the properties of cocoa are required. In this context, this is the first study to assess the effect of plant nutrient balance on the secondary metabolites content of raw fine flavor cocoa bean, variety Arriba Nacional, and the implications for achieving a high antioxidant capacity.

We hypothesized that soil nutrient compositions from different Ecuadorian regions would directly impact the plant nutritional status and thus indirectly affect the antioxidant, metabolomic and organoleptic quality of *Theobroma cacao* L. raw beans, “Arriba Nacional” variety.

## 2. Results

### 2.1. Determination of Methylxanthines (Caffeine and Theobromine)

**Figure 1:**
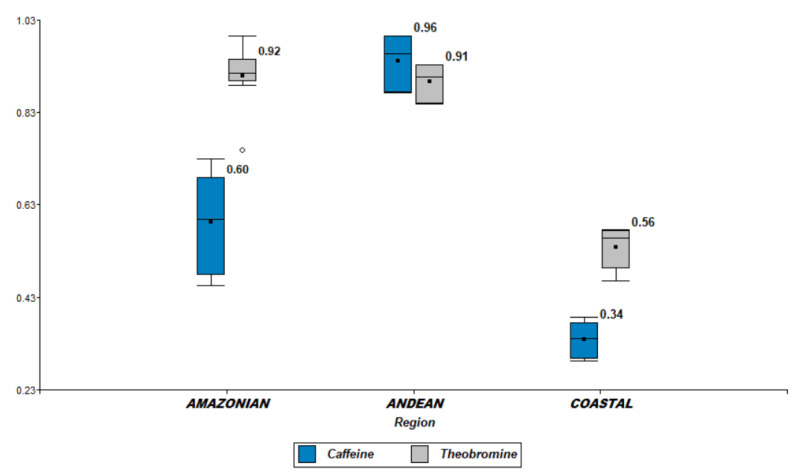
Box plot of methylxanthines (caffeine and theobromine) content with normalized data. The values have been provided to the unit to perform the box plot representation of the different data analyzed.

For caffeine, we observed a higher content (530 ± 40 mg/100 g DW) in the Andean region, followed by the Amazonian region (330 ± 60 mg/100 g DW) and the coastal region (190 ± 23 mg/100 g DW) (nested ANOVA: F_5,12_ = 114.32, *p* < 0.0001, Figure 1). For theobromine, we found a similar trend, with 2540 ± 190 mg/100 g DW in the Amazonian region, followed by the Andean region with 2510 ± 120 mg/100 g DW, and the coastal region with 1500 ± 130 mg/100 g DW (nested ANOVA; F_5,12_ = 50.97, *p* < 0.0001, Figure 1, see Supplementary Materials, Table S1).

### 2.2. Metabolomic Content of Raw Cocoa Beans

**Figure 2:**
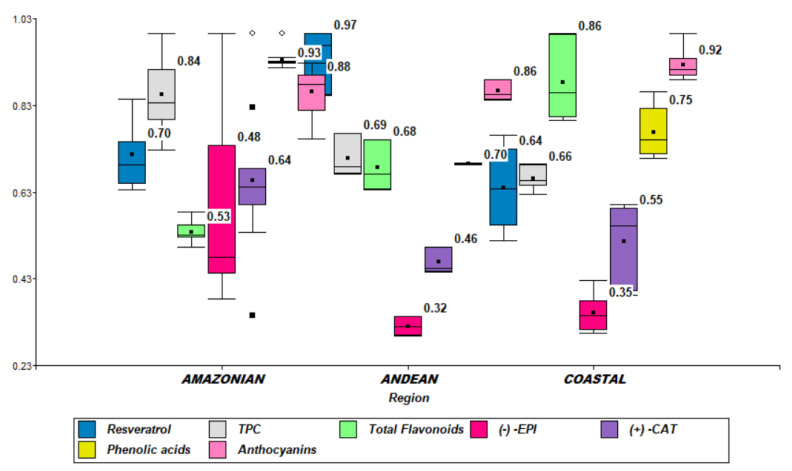
Box plot of the bioactive compounds (resveratrol, TPC—total phenolic content, total flavonoids, (−)-EPI epicatechin, (+)-CAT catechin, phenolic acids and anthocyanins) with normalized data. The values have been provided to the unit to perform the box plot representation of the different data analysis.

The total phenolic content in the research material was determined. The highest phenolic values were found in the beans from the Amazonian region, with 297.00 ± 32.99 mg/100 g of product, followed by those from the Andean region with 246.00 ± 17.42 mg/100 g, whereas the lowest total polyphenolic content with 229 ± 9.78 mg/100 g was found in the beans from the coastal region (Figure 2). The nested ANOVA test (F_5,12_ = 24.05, *p* = 0.0001) showed significant differences in the content of total phenolic content.

The average flavonoid content in cocoa beans from all three Ecuadorian regions ranged from 89 to 308 mg/100 g. The total flavonoid content was the highest in the samples from the coastal region (114.97 ± 12.04 mg/100 g), followed by the samples from the Andean region (89.15 ± 7.68 mg/100 g) and then those from the Amazonian region (69.89 ± 3.16 mg/100 g), following the same trend as that of the total phenolic content (Figure 2). Nested ANOVA (F_5,12_ = 36.07, *p* < 0.0001) showed significant differences between regions.

The total content of anthocyanins in the raw cocoa beans varied among different cocoa groups and ranged from 21 to 22.8 mg/100 g FW, with the highest values registered for the samples from the coastal region (22.80 ± 0.95 mg/100 g FW), followed by the Amazonian region (22.23 ± 1.50 mg/100 g FW). The lowest anthocyanin content was visualized in the cocoa samples from the Andean region (21.28 ± 0.59 mg/100 g FW) (Figure 2). No significant differences were observed between the three regions (nested ANOVA F_5,12_ = 1.1523, *p* = 0.3863).

The highest trans-resveratrol level among the samples in this study was registered in the Andean region (0.1 ± 0.008 mg/100 g). The next highest level of trans-resveratrol (with an average of 0.076 ± 0.008 mg/100 g) could be seen in cocoa samples from the Amazonian region, followed by the Pacific Coast region (0.068 ± 0.010 mg/100 g) (Figure 2). Testing the trans-resveratrol differences among regions, nested ANOVA (F_5,12_ = 22.1843, *p* < 0.0001) showed significant results.

The catechin, representative of the flavan-3-ols group, indicated higher values in the Amazonian region (723 ± 126 mg/100 g), followed by samples from the coastal (568 ± 5 mg/100 g) and Andean regions (515 ± 33 mg/100 g) (nested ANOVA F_5,12_ = 1.6277, *p* = 0.226). The other member of the flavan-3-ols group, epicatechin, showed the same pattern as catechin, with the highest values for the Amazonian region (974 ± 402 mg/100 g) followed by the coastal (590 ± 54 mg/100 g) and the Andean regions (535 ± 37 mg/100 g) (nested ANOVA F_5,12_ = 26.500, *p* < 0.0001-Figure 2).

The occurrence of phenolic acids in cocoa beans from the Amazonian region was reported in higher concentrations (203.28 ± 6 mg/100 g) than in the coastal (203.28 ± 16 mg/100 g) and Andean (183.93 ± 5 mg/100 g) regions (nested ANOVA F_5,12_ = 62.546, *p* < 0.0001-Figure 2, see Supplementary Materials, Table S2).

### 2.3. Antioxidant Capacity of Nacional Raw Cocoa Bean

**Figure 3:**
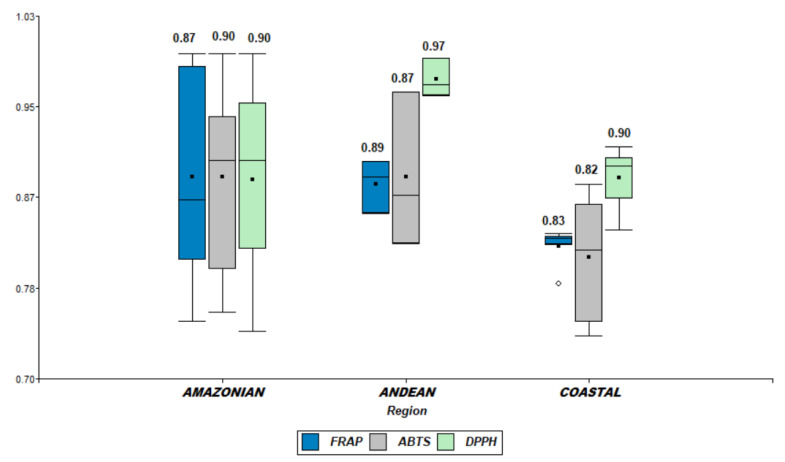
Box plot representation of the antioxidant power (FRAP, ABTS, and DPPH) with normalized data. The values have been provided to the unit to perform the box plot representation of the different data analyzed. Legend: FRAP-Ferric reducing antioxidant power, ABTS-free-radical-scavenging activity, DPPH-α, α-diphenyl-β-picrylhydrazyl free-radical-scavenging method.

Concerning the antioxidant properties, the nested ANOVA test showed no significant differences in the ABTS (F_5,12_ = 1.141, *p* = 0.28), while for DPPH (F_5,12_ = 12.44, *p* = 0.0002) and FRAP (F_5,12_ = 12.57, *p* = 0.0002), differences among the three regions were significant (Figure 3).

Use of the Pearson correlation test to check for relationships among metabolites and antioxidants revealed a positive correlation between FRAP and total phenolic content (Table 1).

### 2.4. DRIS Analysis

Table 2 shows the DRIS indexes of nutrients (according to the area with the higher yield) with the aim to serve as a guide to quickly diagnose the nutrient needs. In the Esmeraldas province of the coastal region, deficiencies were found in the fine flavor cocoa culture, mainly represented by Zn > Cu > Mn micronutrients. From the Amazonian region, the Napo province presented the following deficiency in Zn > Cu; Orellana province in Zn > Cu > Ca; and Sucumbíos in Cu > Zn nutrients. In the Andean region, the Cotopaxi province was characterized by Cu > Zn deficiencies. The nutrients found in excess were represented by K and P in Esmeraldas province from the Pacific Coast region; N, K, and Mn in the Napo and Orellana provinces; N and Mn in Sucumbíos province from the Amazonian region; and N, K, and P for Cotopaxi province, a representative of the Andean region. Both excesses and deficiencies of nutrients influence the synthesis of secondary metabolites in cocoa cultivars.

### 2.5. Multivariate Analysis

Two PCs explained up to 100.0% of the total variance. The first PC (PC1) accounted for 64.1% of the total variability and had positive loadings in terms of flavonoids (0.27) and anthocyanins (0.24), as well as from DRIS indexes of P (0.21), Fe (0.27), K (0.26), and Mg (0.26). The second PC (PC2), representing 35.9% of the total variance, was associated with trans-resveratrol (0.35), FRAP (0.14), caffeine (0.32), ABTS (0.17), DPPH (0.36), and theobromine (0.16), as well as a DRIS index of N (0.15), P (0.22), and Cu (0.15).

Cocoa samples from the Amazonian region showed negative PC1 scores, associated with higher phenolic content, while the coastal region showed positive PC1 scores, associated with flavonoid and anthocyanin contents. The Andean region was characterized by high resveratrol and methylxanthine contents (theobromine and caffeine), as well as high antioxidant activity (positive PC2 scores).

**Figure 4:**
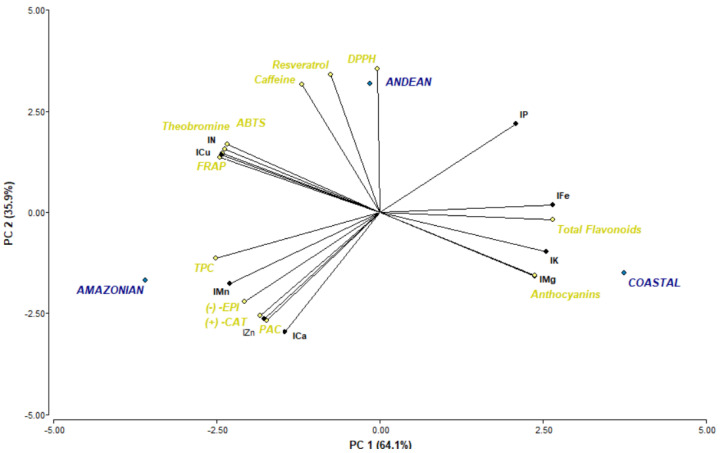
Phytochemical and biological composition of Arriba Nacional cocoa grown in the main Ecuadorian regions, characterized by different nutrient ratios. Loading plot of PC1 versus PC2, showing the correlation with theobromine, caffeine, trans-resveratrol, total phenolic content, total flavonoids, anthocyanin, (−)-epicatechin, (+)-catechin, phenolic acids, FRAP, ABTS, DPPH, and micro- and macronutrients (DRIS indexes) (IN, IK, IP, ICa, IMg, IFe, IMn, IZn, and ICu).

The PCA indicated that the DPPH scavenging activity in the Andean region was correlated with the caffeine and resveratrol content and a conversely stronger association was observed between the ABTS, FRAP antioxidant capacity, N and Cu elements, and theobromine. Anthocyanin content in the Pacific Coast region was correlated with nutrients such as Mg and K, while the flavonoid content was influenced by Fe, Ca, and P content (Figure 4).

### 2.6. Sensory Attributes

No significant difference was found in the raw bean samples from the three different regions for acidity (one-way ANOVA F_2,87_ = 0.937, *p* = 0.396) and sweetness (one-way ANOVA F_2,87_ = 0.102, *p* = 0.903), which ranked as low perception. For “Aroma”, by one-way ANOVA (F_2,87_ = 33.908, *p* < 0.0001), the cocoa samples from the Amazonian region obtained a significantly (Tukey test *p* < 0.0001 for both) higher score than the Andean and coastal samples, which presented similar scores. Additionally, differences were found regarding the astringent (F_2,87_ = 28.65, *p* < 0.0001) and bitter taste (F_2,87_ = 19.70, *p* < 0.0001). The coastal samples had the lowest value, while Amazonian and Andean samples expressed similar scores for each attribute (Table 3).

## 3. Discussion

### 3.1. Determination of Methylxanthines (Caffeine and Theobromine)

Cocoa beans and their derived products contain considerable amounts of methylxanthines (caffeine and theobromine), a group of bioactive secondary cocoa metabolites [13]. Some of the effects of methylxanthines are represented by central nervous system stimulation, diuresis, and cardiovascular and metabolic effects [14]. Nowadays, it is known that theobromine consumption may be able to activate thermogenesis and prevent obesity and other metabolic disorders [15]. Theobromine is accumulated in higher quantities than caffeine in cocoa beans [16], which can explain the higher values of theobromine compared with caffeine in all investigated samples originated from the three Ecuadorian regions. Our results are in accordance with other studies, where theobromine was present in higher concentrations than caffeine in the cocoa beans [17,18].

At the same time, the theobromine and caffeine concentrations in cocoa beans determine the bitter flavor, which is also demonstrated in our experiment, inducing the stronger bitterness found in cocoa samples from the Amazonian and Andean regions. There are different theories about the role played by alkaloids in cocoa. The most solid argument is that their accumulation implies a biological role as defense chemicals, since they act as antiherbivore and allelopathic compounds [19], and especially since the cocoa plant is continuously exposed to the attack of various fungi that produce blackspot, moniliasis, and witch’s broom, among other phytosanitary problems. The Ecuadorian regions that accumulate high amounts of alkaloid content in cocoa beans are exposed to high precipitation, which could exacerbate the fungal attack that triggers the synthesis of these defense compounds to guarantee the protective effect. Methylxanthines are also thought to be present due to excess nitrogen, as increased methylxanthine content has been reported in germination grains [20]. In the case of the Amazonian and Andean regions, this excess N was demonstrated by our DRIS analysis.

### 3.2. Metabolomic Content of Raw Cocoa Beans

The principal raw material for the manufacture of chocolate and cocoa-derived products is cocoa seeds [8]. Global cocoa consumption is increasing due to evidence from clinical and epidemiological studies that suggests that the regular consumption of cocoa-derived products can contribute to preventing chronic illnesses such as cardiometabolic diseases, cancers, and neurodegenerative diseases [21]. These health benefits have been attributed to the occurrence in cocoa beans and cocoa-derived products of polyphenols [22], which constitute approximately 6–8% of the solid substance of cocoa beans.

Cocoa is particularly interesting from a nutritional and health point of view due to its high content of polyphenols [23], which have received considerable attention due to their physiological functions, including antioxidant, antimutagenic and antitumor activities. In our study, we identified significant differences between phenols from different regions. Similar results were obtained for raw cocoa bean hull and cocoa nibs of different origins [18].

The most abundant class of polyphenols identified in cocoa beans is flavonoids [9]: monomeric flavan-3-ols (37%), (−)-epicatechin and (+)-catechinprocyanidins (58%) [24], anthocyanins (4%), low amounts of flavanols (quercetin and its glycosides), and flavones. Cocoa beans contain a number of other non-flavonoid compounds, including phenolic acids, hydroxycinnamic acid amides, and stilbenes [25]. These bioactive compounds are secondary cocoa metabolites that play a fundamental role in plant protection against UV light, pathogens, parasites, and plant predators [24]. Due to climatic conditions (high humidity) that promote pathogen attacks, the Amazonian and Andean regions present a higher content of these metabolites.

### 3.3. Antioxidant Capacity of Nacional Raw Cocoa Bean

The interest in cocoa has increased over time, due to the potential effects on human health induced by its antioxidants. The high antioxidant properties of raw cocoa beans and its flavonol-rich products are connected to the amount of flavon-3-ols ((−)-epicatechin and (+)-catechin), oligomeric and polymeric procyanidins [10], anthocyanins, flavonols (quercetin aglycone and its glycosides), flavones, phenols, phenolic acids, and *trans*-resveratrol [10]. It is known that many flavonoids exhibit high antioxidant activity, due to scavenging free radicals, chelating metal ions, binding proteins, and inhibiting enzymes that generate superoxide radicals [26].

The flavonoids, anthocyanins, and stilbenes (trans-resveratrol) could be observed in higher concentrations in the Arriba Nacional cocoa beans originated from the Amazonian and Andean regions, determining a higher antioxidant activity of these cocoa beans compared with those from the coastal region.

### 3.4. DRIS

The Diagnosis and Recommendation Integrated System (DRIS) is a widely used statistical approach for the interpretation of plant tissue analysis data. It helps in simultaneously identifying imbalances, deficiencies, and excesses of crop nutrients, and ranking them in order of their importance for their remedial steps.

The ecoclimatic and nutritional conditions in the Ecuador territory are conducive to an increased ability to produce cocoa with different phenolic bioactive profiles, and contents that depend on a number of factors, such as the genotype, climatic and agronomic conditions, post-harvest practices, and storage conditions [9].

Various genetic, ontogenic, morphogenetic, and environmental factors can influence the biosynthesis and accumulation of secondary metabolites. Cocoa production of secondary metabolites can be gradually generated in response to environmental stress, and hence plant secondary metabolism can be viewed as a plant behavior that is in part the ability of a plant to adapt and survive in response to environmental stimuli during its lifetime [27].

In our study, the DRIS analysis allowed for the diagnosis of the real nutritive state of cocoa plant characteristics for each of the three Ecuadorian regions. The multivariate analysis emphasized how nutrient differences affect and interfere with cocoa’s secondary metabolites and antioxidant characteristics.

The fine cocoa flavor cultures analyzed in this study generally presented Zn, Cu, Mn, N, and Ca deficiencies in the case of Orellana province (Amazonian region). These nutrients are not available due to various factors, such as pH, since the majority of the soil has a pH that is between slightly and moderately acidic.

The coastal region was correlated in the multivariate analysis with the flavonoids and anthocyanin compounds, and DRIS analysis indicated an excess of Mg, Fe, P, and K, and a deficiency of Zn, Cu, Mn, and N in cocoa plants from this area, elements that are proven to be related to this type of secondary metabolite.

Slightly increased anthocyanin concentrations (between 15% and 70%) in plants are determined by magnesium treatment. Its effect is stronger under high temperature regimes, such as in the case of the coastal area, which had the highest temperatures among the considered regions [28].

The anthocyanin’s stability depends on intra-vacuolar conditions (pH, co-pigmentation with colorless flavonoids, and the formation of complexes with metal ions) [29].

The stress of nitrogen deficiency allows the highest flavonoid content in cocoa beans originating from this area to be obtained, but not accompanied by an increase in the main phenolic compounds [30].

Ca^2+^ helps in the upregulation of the genes involved in the biosynthesis of polyphenols either phenolic acids or flavonoids (flavan-3-ols), which induce resistance against biotic and abiotic stresses [31].

Deficiency of Zn, which follows nitrogen in importance, as in the case of cocoa plants from the Amazonian region, triggers an increase in the synthesis of flavonoids, in particular, the flavan-3-ols (+)-catechin and (−)-epicatechin [32].

### 3.5. Multivariate Analysis

The Amazonian region, with cocoa plants characterized by Mn excess (as revealed by our DRIS analysis), is also represented by a high phenolic composition, and this correlation is proved by the multivariate analysis.

Multiple steps in the biosynthesis of secondary metabolites such as phenolic compounds (phenolic acids and flavon-3-ols) require Mn [33].

The enhancing of phenolic biosynthesis in the Mn-treated plants leads to an increase in plant resistance to the high levels of Mn, due to the role of these compounds in the antioxidant defense of plants in response to environmental factors (such as heavy metals) [34].

DRIS analysis also revealed that cocoa beans from the Andean region present excessive values of N and P nutrients. The multivariate analysis presented a strong correlation between the methylxantines (theobromine and caffeine), the level of N, and antioxidant activity.

The nitrogen level has effects on different aspects of the plant (growth, development, and the metabolic pathways of plants) [35]. Nitrogen content may lead to the promotion of metabolic pathways involving alkaloids [36].

At the same time, theobromine content is correlated with an antioxidant activity determined by FRAP and ABTS methods. Theobromine acts through the inhibition of phosphodiesterases, blocking the receptors of adenosine and reducing cellular oxidative stress (effects independent of adenosine receptor), proving in this way the potent antioxidant effect [37].

Additionally, in the same multivariate analysis could be observed the interdependency of antioxidant activity by DPPH assay and the resveratrol and caffeine composition. We can explain the strong correlation between antioxidant activity and these two compounds (proven antioxidants) with reference to their structures. In the case of resveratrol, an abstraction of a hydrogen atom from the monophenolic hydroxyl group may occur easily [38]. Caffeine is structurally similar to uric acid, and this could explain the antioxidant and pro-oxidant properties of this compound [39].

### 3.6. Sensory Attributes of Raw Cocoa Beans

Throughout history, cocoa has represented a valuable economic and social item in different cultures of the American continent, being a symbolic product within the Inca culture, in which fulfilled consumption and trade functions were used as a currency between aboriginal communities. Nowadays, varieties of cocoa such as fine aroma cocoa represent a desirable product with significant acceptance in different continents, thanks to its chemical and sensory benefits. The present study carried out a sensory characterization of the raw bean of fine aroma cocoa variety, focusing on attributes such as astringency, bitterness, acidity, and sweetness. The panelists carrying out the sensory analysis could not perceive differences in the sweetness and the acidity attributes of the tested samples, and also offered low values. It is possible that compounds such as monosaccharides, disaccharides, oligosaccharides, and some L-amino acids, present in lower concentrations in cocoa beans, contribute to the sweet taste [40].

The same explanation may be proposed for the lack of acidity perception, it being known that the carbohydrate fermentation reactions generate acids, alcohols, and ketones and cause the acidification of the cocoa beans, a process that normally takes place during fermentation, not in the raw material [41].

The bitter taste of cocoa beans is mainly due to the presence of methylxantine compounds (theobromine and caffeine). The taste of unfermented fine flavor cocoa beans is very susceptible to these two compounds. The bitter note of cocoa is correlated with theobromine and caffeine content penetration [17] and explains in our experiment the highest bitterness values of the cocoa samples from the Amazonian and Andean regions, where the content of alkaloids was higher than in the cocoa beans from the coastal regions.

The bitterness of cocoa beans is mainly due to the presence of alkaloids and other molecules (diketopiperazines, free L-amino acids, or peptides) [40]. Tannins (epicatechins, catechins, and procyanidins) are molecules that may contribute to the perception of bitterness as a “bitter-astringent” sensation. Tannins, together with total polyphenols, were found in high concentrations in our samples from the Amazonian and Andean regions.

The astringent taste is associated with the polyphenol-protein interaction in the saliva and is perceived as a dry feeling in the mouth [42]. The strongest sensations of astringency are induced by cocoa beans from the Amazonian and Andean regions, compared with those from the coastal region.

Raw cocoa is known to have an astringent taste and flavor. Unfermented cocoa beans do not contain the aroma precursors that are present in the fermented cocoa beans [43]. In the case of the raw material, the flavor was not so evident for the panelists.

The investigated regions exert different effects on the chemical characteristics and sensory properties exhibited by the cocoa beans.

## 4. Materials and Methods

### 4.1. Chemical Reagents

Ultrapure water was obtained from a Milli-Q water purification system (Millipore Corp., Bedford, MA, USA). The standards of resveratrol (≥99%), (+)-catechin (≥99%), (−)-epicatechin (≥98%), theobromine (≥99%), caffeine (≥99%), quercetin (≥95%), gallic acid, cyanidin-3-glucoside chloride (≥95%), and chlorogenic acid (≥95%) were obtained from Sigma-Aldrich (St. Louis, MO, USA). ABTS (2,2-azinobis-3-ethyl-benzothiazoline-6-sulfonic acid), DPPH (1,1-diphenyl-2-picrylhydrazyl), TPTZ (2,4,6-tris(2-pyridyl)-(S)-triazine), TROLOX (6-hydroxy-2,5,7,8-tetramethylchroman-2-carboxylic acid), Folin–Ciocalteau’s reagent, and ferrous chloride were also purchased from Sigma-Aldrich Chemical Co. (St. Louis, MO, USA). All chemicals were of analytical and HPLC grade and reagents were prepared according to standard analytical procedures.

### 4.2. Plant Material and Sample Collection and Preparation for Phytochemical and Antioxidant Analysis

**Figure 5:**
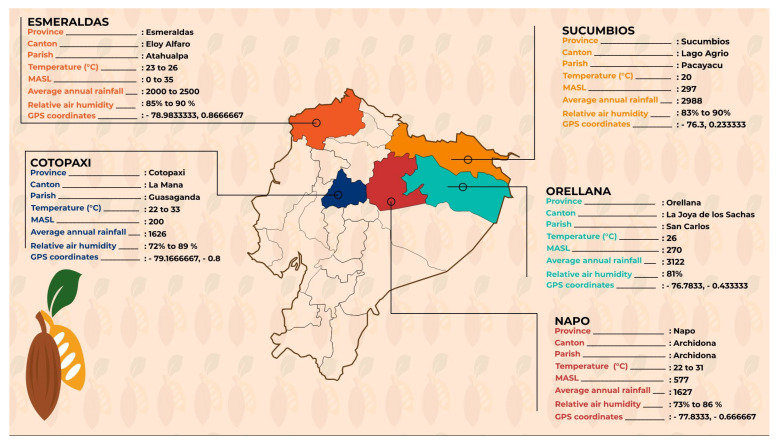
Ecuadorian cocoa-growing areas under study, with a few characteristics.

Mature cacao pods of the cocoa variety “Arriba” were collected in June 2021 from selected farms from three distinct Ecuadorian regions, two representative of the main Ecuadorian cocoa growing areas (the Pacific Coast and the Amazonian region) and one (the Andean region) representing a new promising area for cocoa cultivation. The cocoa-growing area of Ecuador corresponding to the Pacific Coast was represented by the Esmeraldas province, which accounts for a high proportion of the national production. For the Amazonian region, we selected the Sucumbios, Francisco de Orellana and Napo areas (representing 6% of production at the national level) (Ministry of Agriculture and Livestock 2016). Finally, the Andean region was represented by the Cotopaxi Province, a new area investigated for this type (Figure 5). For each province, we selected 3 farms according to the lists of producers provided by local cocoa organizations, and in each farm, we collected samples of 10 cacao fruits, randomly selected from 10 cacao trees (1 fruit/tree), in order to obtain approximately 1 kg of raw cocoa beans per province. The harvested cacao fruits, of each sample, were placed in cooler bags and transported directly to the CICTE laboratory of Universidad de Las Fuerzas Armadas for further analysis.

All the experiments conducted in this research involved the use of raw cocoa beans, with the mucilage removed, from mature cacao pods (in the optimal ripeness stage), avoiding in this way any possible alteration in secondary metabolite compositions. The combination of acidic conditions and high temperatures from cocoa processing (drying and fermentation) lead to major changes in the beans. For example, polyphenols are particularly affected by these transformations, as they are highly reactive molecules.

### 4.3. Preparation of Cocoa Extracts (CE)

To achieve the maximum extraction efficiency during the sample preparation, the cocoa beans were processed using a procedure reported by [44], slightly modified. The raw cocoa bean material was grounded for 50 s in order to obtain a homogeneous material that was defatted four times with 125 mL of hexane for 20 min at 200 rpm and subsequently centrifuged for 30 min at 4000 rpm. The defatted cocoa sample was then extracted four times with acetone 70% *v*/*v* at a ratio of 1:5, stirred for 3 min, and centrifuged for 15 min at 4000 rpm. The supernatants obtained after centrifugation were filtered with Whatman n°1 filter paper and the organic solvent was removed by evaporation at room temperature for 48 h. Finally, the remaining suspension was lyophilized to obtain a stable cocoa extract.

### 4.4. Determination of Methylxanthines by HPLC-DAD

The degreased powder of cocoa samples was extracted following the procedure of [45]. A sample of 300 mg degreased cocoa was extracted with approximately 90 mL of hot water (80 °C) in a heated bath for 20 min. The solution was then cooled down to room temperature, centrifuged at 3000 rpm, and the supernatant was filtered through a 0.45 µm Millipore filter for further analysis by high-performance liquid chromatography (HPLC).

To perform this analysis, an Agilent 1100/1200 Series HPLC (Santa Clara, CA, USA) instrument, with a binary pump (G1312A), column oven (G1316A), diode array detector (DAD, G1315D), and auto-injector (G1329A), was used, controlled by the Chemstation software (Agilent Technologies, Santa Clara, CA, USA). Separation was carried out using an Agilent Zorbax SB C_18_ column 150 × 3.9 mm, internal diameter, particle size, 4 µm, at a flow rate of 1.4 mL/min and a temperature of 22 °C, using as the mobile phase a 20% (*v/v*) methanol solution in deionized water. For analysis, 20 μL of the samples was injected into the equipment and methylxanthines were monitored using the DAD at 274 nm. Identification of methylxanthines was carried out by comparison with the internal standards of theobromine and caffeine. The obtained equations were as follows: 0.2839x − 0.1117 (R^2^ = 0.9800, *n* = 5, *p* = 0.001211) and 0.6488x + 0.1426 (R^2^ = 0.9951, *n* = 5, *p* = 0.000154), respectively. The results are expressed as grams of compound per 100 g of cocoa extract.

### 4.5. Quantification of Total Phenolic Content (TPC)

The total phenolic contents (TPC) were determined spectrophotometrically according to the methodology described by [46] with few modifications, based on the Folin–Ciocalteau colorimetric method [47]. Phenolic groups are oxidized by phosphomolybdic and phosphotungstic acids in the Folin–Ciocalteau reagent, forming a green-blue complex detectable at 710 nm. Briefly, an aliquot of lyophilized cocoa extract dissolved in solvent extraction was diluted to 5 mL of Milli-Q water and was added to the 1.5 mL Folin–Ciocalteau reagent, and after 5 min of reaction at room temperature (25 °C), 2 mL of a 100 g/L solution of Na_2_CO_3_ was added. The absorbance was measured at 710 nm after 30 min with the spectrophotometer against a blank without extract. Gallic acid was used to obtain a calibration curve with standard solutions within the range of 50–250 mg/L. The results are expressed as mg gallic acid equivalent/100 g of cocoa extract (mg GAE/100 g DW). The obtained equation was expressed as y = 0.0157x + 0.0357, correlation coefficient of the calibration curve was 0.9968 (DL = 0.7643 mg/L, QL = 2.5477 mg/L (5.9701%), *n* = 6, *p* = 3.77 × 10^6^).

### 4.6. Quantification of Total Flavonoid Content (TFC)

The total flavonoid content in extracts was determined spectrophotometrically according to the method based on the formation of a flavonoid–aluminum complex with a maximum absorptivity at 430 nm, known as the AlCl_3_ colorimetric method, or the Dowd method [48]. An aliquot of 1 mL of cocoa extract solution (25–200 µg/mL) was mixed with 0.3 mL of 10% (*v/v*) AlCl_3_ solution in methanol, 0.2 mL (1 M) potassium acetate, and 5.6 mL distilled water. The mixture was incubated at room temperature for 10 min before measuring the absorbance of the reaction mixture at 430 nm. Quercetin (QE) was used as the standard for the calibration curve in the range of 1–100 mg/L. The calibration curve had a correlation coefficient of 0.9991, with a DL = 0.4752 mg/L and QL = 1.5843 mg/L (11.5003%). The obtained equation was y = 0.0167x + 0.0245, *n* = 7, *p* = 8.79 × 10^9^. The results are expressed as mg quercetin equivalents/100 g cocoa extract weight.

### 4.7. Quantification of Total Anthocyanin Content (TAC)

The assessment of total anthocyanin content was carried out by the pH differential method according to AOAC as described by [49]. Absorbance was measured at 510 and 700 nm in buffers at pH 1.0 and 4.5. Pigment concentration is expressed as cyanidin 3-glucoside equivalents using the following formula:

Anthocyanin pigment (cyanidin-3-glucoside equivalents, mg/L) = $\frac{A\times \;\mathrm{MW}\;\times \;\mathrm{DF}\;\times 1000\;}{\varepsilon \times 1}$ where A = (A520 − A700 nm) pH 1.0 − (A520 − A700 nm) pH 4.5; MW (molecular weight) = 449.2 g/mol for cyanidin-3-glucoside (cyd-3-glu); DF = dilution factor established in D; l = path length in cm; (ε = 26.900 molar extinction coefficient, in L × mol^−1^ × cm^−1^, for cyd-3-glu; and 10^3^ = factor for conversion from g to mg).

A Unico 2800 UV-Vis spectrophotometer (Unico Instruments Co. Ltd., Shanghai, China) was used for all the spectrometric measurements.

### 4.8. Quantification of Resveratrol, Flavanols, Flavan-3-ols and Phenolic Acids Content

The resveratrol compound used for HPLC analysis was dissolved in methanol at 1 mg/mL concentration and stored away from direct light at 40 °C. The standard methanolic solution of resveratrol was diluted in a range from 10 µg/L to 10 mg/L to create a calibration curve necessary for trans-resveratrol quantification in cocoa extracts. Cocoa samples, for the determination of their stilbene content, were analyzed as indicated in the methodology by Katsagonis et al., 2005 [50] with some modifications using an HPLC system (JASCO Model AS-2051 liquid chromatography), equipped with Jasco MD2015 Plusmultiwavelenght Detector and the Jasco FP-2020 Plus Intelligent Fluorescent Detector coupled with the PROGRAM Jasco-Chrompass Chromatography Data System. Prior to HPLC analysis, all cocoa extracts were centrifuged at 15,000× *g* for 15 min. Reversed-phase chromatographic separation was conducted on a Nova-Pak C_18_ column with a 4 μm particle size, and a 150 mm × 4.0 mm internal diameter column. Elution was performed with a linear gradient of 0–95% in 20% HPLC-grade acetonitrile for 35 min with a flow rate of 1 mL/min. The eluent was monitored at 312 ± 2 nm, which is the maximum UV absorbency of *trans*-resveratrol [51]. The used linear regression equation was y = 400.628x − 144.146, R^2^ = 0.9998, *n* = 3, *p* = 0.001, DL = 1.29 μg/mL, QL = 4.29 μg/mL.

The detection of the other bioactive compounds was performed at 280 nm in the case of flavan-3-ols and 320 nm for the phenolic acids, respectively. (−)-epicatechin, (+)-catechin, and chlorogenic acid were used as external standards for the quantification of flavan-3-ols and phenolic acid, respectively. The results are expressed in mg/100 g of degreased cocoa extract [52]. Calibration curves were obtained for (−)-epicatechin, (+)-catechin and phenolic acids. The regression of calibration curves was as follows 1.2861x + 0.1618 (R^2^ = 0.9785, *n* = 4, *p* = 0.009521), 1.2926x + 0.8864 (R^2^ = 0.9917, *n* = 5, *p* = 0.00135) and 0.0192x − 0.0188 (R^2^ = 0.9807, *n* = 5, *p* = 0.001293), respectively.

### 4.9. Evaluation of Antioxidant Activity by the ABTS Method

The ABTS free-radical-scavenging activity of each sample was determined according to the method described by [53]. The ABTS radical cation was produced by reacting ABTS with potassium persulfate. A mixture of ABTS (2 mM) and potassium persulfate (70 mM) was allowed to stand overnight at room temperature in the dark to form the radical cation ABTS, 16 h prior to use. The ABTS solution was then diluted with 80% methanol to obtain an absorbance of 0.700 ± 0.005 at 734 nm. A total of 100 μL of appropriately diluted samples was added to 2 mL of ABTS solution and the absorbance was recorded at 734 nm after 1 min of incubation at room temperature. A standard curve was obtained by using TROLOX standard solution at various concentrations (ranging from 0 to 0.24 μg/mL). The TROLOX calibration curve of tested solutions was constructed in the range 10.0–150.0 mg/L. The linearity for ABTS was expressed by the equation y = −0.4202x + 0.7797, (R^2^ = 0.9903, *n* = 8, *p* = 0.00102), DL = 0.01796 mg/L, and QL = 0.05989 mg/L (0.3250%). The scavenging activity of cocoa extracts and fractions against ABTS radicals was also measured to calculate IC_50_.

### 4.10. Free-Radical-Scavenging Ability by the Use of a Stable DPPH^•^ Radical

DPPH is a stable free radical which shows a red-purple color in methanol solution, and has a maximal absorption at 515 nm. This assay is based on the decoloration of the DPPH free radical solution due to the free-radical-scavenging effect of antioxidants [54]. The DPPH^•^ radical-scavenging activity was determined using the method fixed reaction time presented by [55]. Briefly, 50 µL of processed MeOH extract was added to 2 mL of fresh 0.1 mM solution of DPPH in methanol and allowed to react at room temperature in the dark. After thirty minutes, the absorbance was measured at 517 nm. The DPPH scavenging ability as a percentage was calculated as: DPPH scavenging ability = (A_control_ − A_sample_/A_control_) × 100. Afterward, a curve of % DPPH bleaching activity versus concentration was plotted and IC_50_ values were calculated. IC_50_ denotes the concentration of sample required to scavenge 50% of the DPPH free radicals. The lower the IC_50_ value, the more powerful the antioxidant activity. The TROLOX calibration curve of tested solutions was constructed in the range 10.0–100.0 mg/L. Calibration curve was expressed by the equation y = 0.9081x + 0.08, R^2^ = 0.9542, *n* = 7, *p* = 0.000151), DL = 0.00809 mg/L and QL = 0.02698 mg/L (0.2970%).

### 4.11. Ferric Reducing Antioxidant Power (FRAP) Assay

The method uses antioxidants as reductants in a redox-linked colorimetric method, employing an easily reduced oxidant, ferric ion (Fe^3+^), present in stoichiometric excess [56]. At low pH, the ferric tripyridyltriazine (Fe^3+^-TPTZ) complex is reduced by antioxidants in the sample to the ferrous form (Fe^2+^-TPTZ), which appears as an intense blue color. The reaction can be monitored at 593 nm. Briefly, the FRAP reagent was prepared by mixing acetate buffer (300 mM, pH 3.6), a solution of 10 mM TPTZ in 40 mM HCl, and 20 mM FeCl_3_ at 10:1:1 (*v*/*v*/*v*). The sample was incubated at 37 °C with 2 mL of the FRAP solution (prepared by mixing 25 mL acetate buffer, 5 mL TPTZ solution, and 10 mL FeCl_3_·6H_2_O solution) for 30 min in the dark. The absorbance of the blue ferrous tripyridyltriazine complex formed was then read at 593 nm. All solutions were used on the day of preparation. For each extract, three replicates were analyzed. The calibration curve was constructed using six calibration solutions of ferric sulfate (Fe_2_SO_3_) in the range 0.10–1.00 mmol/L. Calibration curve was expressed by the equation y = 1.5431x + 0.0004, R^2^ = 0.9961, *n* = 7, *p* = 3.29 × 10^7^), DL = 0.005939 mmol/L, QL = 0.01979 mmol/L (0.3740%).

### 4.12. Plant Material and Sample Collection for DRIS Analysis

#### 4.12.1. Foliar Sample Collection

Foliar samples were collected from selected farms in the morning hours. For each sample, we took 15 subsamples, made up of 4 leaves from the middle third of the plant, to obtain 60 leaves per sample, distributed on the four opposite sides of the plant. All leaves had recently maturated.

Samples consisted of visible dewlap (TVD) leaf blades and were transported in paper bags in cooler containers to the laboratory, where they were wiped clean and the midribs separated and discarded before drying the laminae at 70 °C. The dry leaf material was grounded in a stainless-steel Wiley mill and analyzed for total N by micro-Kjeldahl. The leaf samples were prepared and analyzed through atomic absorption spectrophotometry for K, Ca, Mg, Fe, Zn, and Cu elements, a colorimetric method using vanadate molybdate reaction for P, a turbidimetric method for Bo, and photometry for S. The macronutrients (expressed in percentage) and the micronutrients (mg/kg) were compared with the standards developed by Marrocos et al., 2020 [57].

#### 4.12.2. Soil Sample Collection

The soil samples (collected in the morning hours) consisted of 10 cores to a depth of 30 cm and were transported in plastic bags disposed in cooler recipients to the laboratory, where they were analyzed for pH, P, K, Ca, and Mg by the standard procedures of the Agricultural Research and Education Center (AREC), Belle Glade. Soil pH was determined in a 1:2 soil–water suspension; P was extracted with deionized water and determined colorimetrically; and K, Ca, and Mg were extracted with 0.5 N acetic acid and determined by atomic absorption spectrophotometry. A portion of the ground sample was digested on an AI block with nitric and perchloric acids [58]. Total P was determined by the molybdovanadophosphate colorimetric procedure [59] and K, Ca, Mg, Fe, Mn, Zn, and Cu by atomic absorption spectrophotometry.

### 4.13. DRIS Analysis

A common method used to diagnose nutritional imbalances is the Diagnosis and Recommendation Integrated System, DRIS [60,61]. DRIS is an appealing diagnostic method that integrates dual ratios and functions into nutrient indices and arranges nutrients in the order of their limitation to produce the yield [60]. Dual ratios used to compute DRIS functions are as follows [60]:

$$\mathrm{Index}\;A=\frac{[f\left(A/B\right)+f\left(A/C\right)+f\left(A/D\right)\ldots \ldots +f\left(A/N\right)]}{Z}\;\mathrm{Index}\;B=\frac{\left[-f\left(A/B\right)+f\left(B/C\right)+f\left(B/D\right)\ldots \ldots +f\left(B/N\right)\right]}{Z}\;\mathrm{Index}\;N=\frac{\left[-f\left(A/N\right)+f\left(B/N\right)-f\left(C/N\right)\ldots \ldots +f\left(M/N\right)\right]}{Z}$$

where *A*/*B* and *a*/*b* are dual nutrient ratios in diagnosed and reference compositions, respectively, and *cv* is the coefficient of variation (standard deviation divided by mean) of the reference dual ratio. Factor *κ* accounts for differential measurement units. The ratio expressions (*X*/*Y* or *Y*/*X*) are selected from the highest variance ratio between the low- and high-yielding subpopulations.

The DRIS indices *I_A_*, *I_B_*, and *I_C_* are computed across nutrients *A*, *B*, and *C* by averaging DRIS functions after multiplying DRIS functions by (+1) if the nutrient is at the numerator, or (−1) otherwise, as follows [60]:

$$ƒ\left(A/B\right)=\frac{\left(A/B-1\right)}{a/b}\;\times \;\frac{100}{CV}$$

$$ƒ\left(A/B\right)=\frac{\left(1-A/B\right)}{a/b}\;\times \;\frac{CV}{100}$$

where *NII* is the nutrient imbalance index. The DRIS indices are symmetrical, so their sum is constrained to zero [62].

The DRIS was claimed to diagnose nutrient status irrespective of plant age and location [63].

### 4.14. Sensory Evaluation of Row Cocoa Beans

#### 4.14.1. Selection and Training of Panelists

The selection of 60 panelists (equality of gender, with an average age of 47 years old) was made based on the procedures of descriptive analysis (QDA method) [64], through a questionnaire administered to assess health status, eating habits, interest in participating, no allergic reactions to the consumption of cocoa and its derivatives, and the ability of panelists to express the taste sensations caused by tasting a derivative of cocoa.

Detection tests were carried out to familiarize panelists in sessions of 2 h per week for 2 weeks, with characteristic descriptors of taste, using solutions of sucrose (for the sweet taste), of caffeine (for the bitter taste), of citric acid (for the acidic taste), and of tannic acid for the astringent attribute (International Organization for Standards, 1993). The sixty candidates were considered acceptable when correct in 100% of the tests.

#### 4.14.2. Sample Preparation and Sensory Evaluation

Before the evaluation, 100 g of raw beans from each representative group of the Amazonian, Andean and coastal regions was cleaned, the rind was removed, and the beans were ground into small pieces. A total of 30 sealed plastic containers were prepared for taste and aroma, each one with a content of 1.5 g of ground beans, modified from [65]. The containers were sealed and left for 6 h at a temperature of 28 °C to promote the accumulation of volatiles. The final evaluation was conducted in a single session in an enclosed area with adequate lighting and a temperature of 25 °C.

Panelists were asked to evaluate the samples for sensory attributes based on a quantitative assessment scale following a hedonic scale of 10 points, according to their rating (0 = not perceived at all, 1–2 = low, 3–5 = medium, 6–8 = high, 9–10 = highly perceived) [66]. The evaluated sensory profiles were limited to basic flavors (acidity, bitterness, astringency, and sweetness) and aroma, following the description provided in [67].

For the sensory analysis, 10 samples of each of the treatments after storage for 5 days were evaluated by the 60 consumers, expressed by means ± SE (standard error).

### 4.15. Statistical Analysis

To analyze the antioxidant capacity parameters of the extracts, three replications were used, and the results are expressed by means ± SD (standard deviation).

Given the study design with multiple areas for sampling, nested ANOVA was performed to analyze the differences between groups of metabolomic content and antioxidant capacity, according to the regions and farms studied.

In the nested ANOVA, we had three replicates for each farm (Napo, Orellana, Sucumbios, Esmeraldas, Esmeraldas 2, and Cotopaxi), and the factor Farm was nested in the factor Region (Amazonia, coastal, and Andean).

Additionally, one-way ANOVA analysis was applied to test differences in sensory attributes.

The post hoc Tukey test for multiple comparisons was used to determine the significantly different groups in the ANOVA analysis [68].

Correlations between secondary metabolites (TFC, TPC, TAC, and resveratrol) and antioxidant activity (ABTS, DPPH, and FRAP) were tested using Pearson correlation tests. Analyses were carried out using XLSTAT [69].

A PCA was applied to separate the samples using values of different metabolites (theobromine, caffeine, total phenolic compounds, flavonoid content, total anthocyanins, phenolic acids, and flavan-3-ols) and antioxidant power (DPPH, ABTS, and FRAP). All data were normalized to perform the analysis, where each column data matrix was mean-centered and scaled to unit variance to avoid the effect of different scales of the variables. PCA was conducted using Info Stat/L [70].

## 5. Conclusions

This investigation outlined a general picture of the abiotic factor soil nutrient composition, responsible for the plant nutritional status and the synthesis of valuable secondary metabolites of the “Arriba Nacional” cocoa variety-one of the most appreciated varieties in the world.

In our case, soil nutrient compounds such as Mn, Fe, N, P, Ca, Zn, and Cu were involved in the bioactive and sensory attributes of this cocoa variety. Cocoa beans from Coastal areas showed an increase in anthocyanin content correlated with an excess of Mg in the soil. The nitrogen deficiency induced the highest flavonoid and phenolic contents in cocoa beans. Ca^2+^ ions, which were involved in the biosynthesis of flavonoids, and Zn deficiency were correlated with increased flavonoid content.

The involvement of the abiotic factor, the soil nutritional composition, in the synthesis of cocoa bean secondary metabolites offers the possibility to manage these nutrients by fertilization and to produce cocoa beans of high value. These beans can be processed into industrial products and used to generate new pharmaceuticals, nutraceuticals, or functional foods with consistent quality and a high quantity of bioactive compounds.

## Figures and Tables

**Table 1:** Pearson correlation matrix between 349 metabolites (resveratrol, TPC, TFC, and TAC) and antioxidant properties (ABTS, DPPH, and FRAP). The correlation between TPC and FRAP resisted the Bonferroni correction.

Variables	Resveratrol		TPC		TFC		TAC	
	*r*	*p*	*r*	*p*	*r*	*p*	*r*	*p*
ABTS	0.2852	0.2513	0.2916	0.2404	−0.2436	0.33	−0.2654	0.2872
DPPH	0.0737	0.7713	0.2766	0.2665	0.0739	0.7708	−0.0837	0.7412
FRAP	0.0587	0.8172	**0.6512**	**0.0034**	−0.412	0.0894	−0.3284	0.1834

Bold values are significant at α ≤ 0.05/12 = *p* ≤ 0.004, *r*-Pearson correlation coefficients and *p*-*p* values. Legend: TPC-total phenolic content, TFC-total flavonoids content, TAC-total anthocyanin content, ABTS-free-radical-scavenging assay, DPPH-α, α-diphenyl-β-picrylhydrazyl free-radical-scavenging method, FRAP-Ferric reducing antioxidant power.

**Table 2:** DRIS indexes for fine flavor cocoa culture (IN, IK, IP, ICa, IMg, IFe, IMn, IZn, and ICu) in different Ecuadorian regions (coastal, Amazonian, and Andean).

Regions/Provinces					
DRIS Index	Coastal		Amazonian		Andean
	Esmeralda	Napo	Francisco de Orellana	Sucumbios	Cotopaxi
IN	20	23.6	22.1	18.6	19.9
IK	49.2	29.1	52.26	2.4	30.1
IP	16.8	0.8	3.7	−1.7	13.5
ICa	−1.2	4.5	−19	0.7	1.4
IMg	8.8	7.7	−4.2	−1.3	1.1
IFe	6.4	−0.6	2.7	−0.1	7.6
IMn	−22.6	79.5	39.8	45	−18.2
IZn	−32.3	−88.6	−57.2	−30.7	−14.5
ICu	−27.2	−49.1	−43.8	−32.9	−41.0

Legend: IN, IK, IP, ICa, IMg, IFe, IMn, IZn and ICu-DRIS indexes of soil nutrients.

**Table 3:** Sensory attributes evaluated.

Sensory Attributes	Treatment			
	Amazonian Region	Andean Region	Coastal Region	*p*
Acidity	1.00 ± 0.79	0.97 ± 0.76	0.733 ± 0.69	*p* = 0.328
Aroma	9.00 ± 0.91	7.07 ± 0.74	7.23 ± 1.22	*p* < 0.0001
Astringency	7.43 ± 1.10	7.40 ± 1.07	5.80 ± 0.81	*p* < 0.0001
Bitterness	7.23 ± 1.07	7.63 ± 1.13	6.07 ± 0.83	*p* < 0.0001
Sweetness	1.03 ± 0.85	0.97 ± 0.81	0.97 ± 0.93	*p* = 0.942

The data are represented by mean ± standard deviation, and the *p*-values of ANOVA.

## Data Availability

Not applicable.

## Supplementary Materials

The following supporting information can be downloaded at: https://www.mdpi.com/article/10.3390/plants11070976/s1, Table S1: The alkaloids compounds (caffeine, theobromine) of the “Arriba Nacional” cocoa variety collected from the three regions studied. Table S2: The antioxidants of the “Arriba Nacional” cocoa variety collected from the three regions studied.
