# Supplementary material for: Abiotic Factors from Different Ecuadorian Regions and Their Contribution to Antioxidant, Metabolomic and Organoleptic Quality of Theobroma cacao L. Beans, Variety “Arriba Nacional”
Source: Plants (Basel). 2022 Apr 3;11(7):976. doi: 10.3390/plants11070976 (PMC9002803; doi:10.3390/plants11070976)
Supplement: Supplementary file 1 [file plants-11-00976-s001.zip › plants-1615542-supplementary.pdf]

Abiotic factors from different Ecuadorian regions and its contribution to antioxidant, metabolomic and organoleptic quality of *Theobroma cacao* L. beans, variety "Arriba Nacional"

Raluca A. Mihai[1a*], Pablo A. Landazuri Abarca[1b], Bryan A. Romero Tinizaray[2], Larisa I. Florescu[3], Rodica D. Catană[3*] and Anna Kosakyan[4]

Table S1. The alkaloids compounds (caffeine, theobromine) of "Arriba Nacional" cocoa variety collected from the three regions studied.

Region	Alkaloids Caffeine (mg/100 g)	Theobromine (mg/100 g)
Amazonia	330 ± 60 S.D.	2540 ± 190 S.D.
Andes	530 ± 40 S.D.	2510 ± 120 S.D.
Coast	190 ± 23 S.D.	1500 ± 130 S.D.

Table S2. The antioxidants of "Arriba Nacional" cocoa variety collected from the three regions studied.

Region	Antioxidants						
	TPC (mg/100 g)	TFC (mg/100 g)	TAC (mg/100 g)	T-resveratrol (mg/100 g)	Catechin (mg/100 g)	Epicatechin (mg/100 g)	Phenolic acids (mg/100 g)
Amazonia	297.00 ± 32.99 S.D.	69.89 ± S.D.	22.23 ± 1.50 S.D.	0.076 ± 0.008 S.D.	723 ± 126 S.D.	974 ± 402 S.D.	249.4 ± 43 S.D.
Andes	246.00 ± 17.42 S.D.	89.15 ± 7.67 S.D.	21.28 ± 0.59 S.D.	0.100 ± 0.008 S.D.	568 ± 5 S.D.	590 ± 54 S.D.	183.6 ± 5 S.D.
Coast	229.71 ± 9.78 S.D.	114.97 ± 12.04 S.D.	22.80 ± 0.95 S.D.	0.068 ± 0.010 S.D.	515 ± 33 S.D.	535 ± 37 S.D.	206.0 ± 64 S.D.

Legend: TPC – total phenolic content, TFC – total flavonoids content, TAC – total anthocyanin content
